# The Payment of Hospital Staffs

**Published:** 1910-09-10

**Authors:** 


### The Hospital

A Journal of

The Medical Sciences and Hospital Administration.

New Series. No. 187, Vol. VII. [No. 1259, Vol. XLVIII.] SATURDAY, SEPTEMBER 10, 1910.

### The Payment of Hospital Staffs.

Those who have followed the British Medical Association's discussion on the Economic Basis of Hospital Management, and the correspondence between Dr. Heron and the Hon. Sydney Holland, to which the report of it in these columns gave rise (The Hospital, August 6, 1910, p. 563), will have observed that on at least one fundamental issue these two gentlemen are wholly at variance. It is usually found to be the case when two men as highly intelligent and as respected as these take diametrically opposite views of some single issue that most people find themselves adopting a line somewhere between that of the two extreme positions, though it may not necessarily be exactly the mean between them. Mr. Holland says that men seek appointments to hospital staffs "because they get a *quid pro quo* for their services. Any man who belonged to the staff of a hospital knew that it was an advertisement for him in his profession, that it was a step up in his profession, and that it enabled him to get patients." Dr. Heron describes this and other remarks of similar tenour spoken at the same time as "insulting to the honorary medical officers of hospitals, and unworthy of a man occupying Mr. Holland's high position in the hospital work of London." That is, in effect, he regards Mr. Holland's analysis of the motives of hospital staffs as inaccurate and misleading.

Now Dr. Heron is a veteran hospital physician whose knowledge of members of hospital staffs in London is probably both larger and more intimate than that of his antagonist. When, therefore, Dr. Heron, whose absolute *bona fides* is entirely beyond question, is moved to the indignation which resulted in his speech at the British Medical Association meeting, there is substantial ground for a close scrutiny of the offending sentences. If the words spoken by Mr. Holland which we have quoted above are carefully considered, it is clear that they are, as they stand, erroneous. Whether men who join a hospital staff expect to get a *quid pro quo* for their services is one thing; whether they actually do get it is quite another. It is said categorically that they do get advertisement, that they do get patients, through belonging to the staff of a hospital. This is an overstatement entirely: there are many men working on the honorary staff of hospitals, not only in London, but in other large towns, who have never yet got one single patient from the connection, and probably never will; and, what is more, they know it. That in the great majority of cases, though even then not in all, the hope or the prospect of thus "getting a step up" in the profession is the motive which induces a man to apply for such a post, may be admitted, though there is usually superadded a motive which is perhaps more altruistic, namely, the desire to perfect himself in his art for the sake of that art alone. This really more exactly represents Mr. Holland's contention, it may be suspected, since he admits, in the paragraph where he compares the barrister's devilling with the consultant's hospital work that many men fail to achieve the success in private practice which they hope for when they join a hospital staff. While Mr. Holland doubtless meant no more than that, he yet laid himself open to Dr. Heron's rejoinder by the sentences quoted above.

Even, however, if Mr. Holland merely means that the hope of professional advancement is the attraction which draws men on to honorary staffs, and that even when that hope is not gratified it is still incorrect to regard the services as given: even then, though he is right about the majority, he is too sweeping and certainly wrong about some. It is only repeating what is fairly common knowledge in the hospital world, that there are a few men whose devotion to their hospital work is quite untinged by *arrière pensée* of personal gain or advancement; and if we never recognised them, it is surely from inability to perceive rather than from lack of opportunity. Probably this trait is better developed among the staff of children's hospitals than anywhere else. It is a commonplace among young consultants that "children's hospitals do not pay"; that is, that no commensurate pecuniary advantage is to be expected from a position on the honorary staff of a children's hospital. Yet there is never any difficulty in filling up vacant posts; indeed, the competition is often very keen for them. This is due to the charm which the work of treating sick children possesses for many medical men; and, whatever may be true of the majority of honorary staffs at other hospitals, it is quite certain that very many of those who work at children's hospitals do so from motives of which sarcastic cynicism takes no cognisance.

Ought, then, hospital staffs to be paid? To that question the answer is, with certain limitations, Yes. Physicians and surgeons should be paid some such sum as will insure them against actual want in the station of life from whose ranks they are drawn. As a set-off against this their appointments should be reviewed at intervals, say every five years; this system already prevails in a few institutions, and is a necessary precaution against the permanent employment of someone who turns out to be a round peg in a square hole. Also pluralism would need regulation; some think that no man should be allowed to serve on the honorary staff of more than one hospital, and if all these posts were paid ones there would certainly be something to be said in favour of the plan. At any rate, the limit would have to be two, even if one should not commend itself. The sum of money necessary to carry out this reform is, of course, enormous; whether too enormous to be practicable is a big question. But the whole body politic, from duke to dustman, has an interest in the solution of this problem. For it is every year increasingly difficult for a young medical man to take up a staff appointment—to become, that is, a budding consultant—unless he has very considerable private means. It is no exaggeration to say that many and many a man is compelled by *res angustæ domi* to settle down into general practice who would have made a most efficient consultant had he been able to take an appointment and face the first few years of hard work and no pay. To pay hospital staffs would mean that the finest intellects in the profession would be secured for this work; at present, for the reasons just given, it is notorious that this is not always the case.

